# Construction of a balanced scorecard-based performance evaluation indicator system for research management in key biomedical research laboratories: An observational study

**DOI:** 10.1097/MD.0000000000048364

**Published:** 2026-05-22

**Authors:** Bing Zhou, Jia Niu, Qi Wang

**Affiliations:** a Department of Endocrinology, NHC Key Lab of Hormones and Development and Tianjin Key Lab of Metabolic Diseases, Tianjin Medical University Chu Hsien-I Memorial Hospital & Institute of Endocrinology, Tianjin, China; b Department of Diagnostic Radiology, Jinling Hospital, Affiliated Hospital of Medical School, Nanjing University, Nanjing, China; c Department of Preventive Healthcare, Chongqing Xinqiao Hospital, No.183, Xinqiao Zhengjie Street, Shapingba, Chongqing, China.

**Keywords:** analytic hierarchy process, balanced scorecard, biomedical research laboratory, delphi method, performance evaluation, research management

## Abstract

The need for robust performance evaluation frameworks in research laboratories has become increasingly critical. This study aimed to develop a comprehensive system for key biomedical research laboratories. To construct a scientifically validated and comprehensive performance evaluation indicator system for research management in key biomedical research laboratories based on the balanced scorecard framework. A mixed-methods approach was employed, integrating literature review, Delphi expert consultation (n = 17), and analytic hierarchy process (AHP). The initial indicator framework was developed through systematic literature analysis and refined through 2 rounds of Delphi consultation. The AHP method was used to determine indicator weights. Consistency was assessed using Kendall coefficient of concordance (*W*) and consistency ratio. The final evaluation system comprises 4 primary indicators (financial, customer/service, internal process, learning and growth), 12 secondary indicators, and 28 tertiary indicators. Expert consensus was achieved with *W* = 0.812 (*P* < .001) after 2 Delphi rounds. The AHP analysis yielded weights of 0.245 for internal process, 0.232 for learning and growth, 0.278 for financial, and 0.245 for customer/service perspectives. The consistency ratio (= 0.089) indicated acceptable consistency. The developed balanced scorecard-based performance evaluation system provides a comprehensive and balanced framework for assessing research management in key medical laboratories, emphasizing strategic orientation and sustainable development.

## 1. Introduction

Biomedical research laboratories play a pivotal role in advancing scientific innovation, translating research discoveries into clinical applications, and cultivating the next generation of biomedical researchers. As healthcare systems worldwide face increasing demands for efficiency and accountability, the need for robust performance evaluation frameworks in research laboratories has become increasingly critical.^[1,2]^ However, current evaluation approaches in medical laboratories often suffer from significant limitations, including overemphasis on single-dimensional metrics, lack of systematic assessment frameworks, and insufficient consideration of nonfinancial indicators that capture the broader impact of laboratory research.^[3,4]^ The traditional performance measurement systems in biomedical research laboratories have predominantly focused on quantitative outputs such as publication counts, grant funding, and patent applications, while neglecting crucial aspects such as knowledge transfer, organizational learning, and stakeholder satisfaction.^[5]^ This narrow focus fails to capture the multifaceted nature of modern medical research laboratories, which must balance scientific excellence with clinical relevance, educational responsibilities, and sustainable resource management.^[6]^ Furthermore, the absence of standardized evaluation frameworks has hindered benchmarking efforts and limited the ability of laboratory managers to identify areas for strategic improvement.^[7]^

The balanced scorecard (BSC), originally developed by Kaplan and Norton for business management, has emerged as a promising framework for addressing these limitations in healthcare organizations.^[8]^ The BSC approach transcends traditional financial metrics by incorporating 4 interconnected perspectives: financial, customer/service, internal business processes, and learning and growth.^[9]^ This multidimensional framework has been successfully implemented in various healthcare settings, demonstrating its versatility in capturing both tangible and intangible aspects of organizational performance.^[10,11]^ Studies have shown that BSC implementation in clinical laboratories can enhance strategic alignment, improve resource allocation, and facilitate continuous quality improvement.^[12,13]^

Recent applications of BSC in healthcare have yielded significant insights into its potential for laboratory management. A systematic review by Bohm et al (2021) found that BSC implementation in healthcare settings improved organizational performance across multiple dimensions, with particular benefits in strategic planning and performance monitoring.^[11]^ Similarly, research by Pham et al (2020) demonstrated that BSC-based evaluation systems enhanced transparency and accountability in hospital laboratories while promoting evidence-based decision-making.^[14]^ The framework’s ability to balance short-term operational goals with long-term strategic objectives makes it particularly suitable for research-intensive medical laboratories that must navigate complex stakeholder expectations and resource constraints.^[15]^

Despite these promising developments, the application of BSC specifically to research management in key biomedical research laboratories remains underexplored. The unique characteristics of medical research laboratories, including their dual role in scientific discovery and clinical service, necessitate a tailored approach that adapts BSC principles to this specific context.^[16]^ Furthermore, the integration of emerging technologies, evolving regulatory requirements, and increasing emphasis on translational research present additional challenges that must be addressed in contemporary performance evaluation frameworks.^[17,18]^ The theoretical significance of this research lies in its contribution to the integration of strategic management tools with scientific research performance evaluation. By adapting the BSC framework to the specific context of medical laboratory research management, this study bridges the gap between business management theory and healthcare administration practice.^[19]^ From a practical perspective, the development of a comprehensive evaluation framework provides laboratory directors and policymakers with evidence-based tools for resource allocation, strategic planning, and performance improvement.^[20]^ The primary objective of this study is to construct a scientifically rigorous, operationally feasible, and strategically aligned performance evaluation indicator system for research management in key medical laboratories based on the 4 BSC perspectives. This framework aims to: provide a balanced assessment incorporating both financial and nonfinancial metrics, facilitate strategic alignment between laboratory operations and institutional objectives, support evidence-based decision-making in resource allocation and research prioritization, and enable continuous performance monitoring and improvement.

## 2. Methods

### 2.1. Research design

This study employed a comprehensive mixed-methods approach to develop and validate a BSC-based performance evaluation indicator system for biomedical research laboratory management. The research design integrated qualitative and quantitative methodologies to ensure both theoretical rigor and practical applicability. The overall framework combined systematic literature review for theoretical foundation, Delphi expert consultation for content validation and refinement, and analytic hierarchy process (AHP) for quantitative weight determination. This triangulated approach was selected to leverage the complementary strengths of each method while mitigating their individual limitations.^[21]^ The research was conducted between January 2024 and December 2024. This study was approved by the Ethics Committee of Chongqing Xinqiao Hospital. All procedures performed in this study involving human participants were in accordance with the ethical standards of the institutional and/or national research committee and with the 1964 Helsinki declaration and its later amendments or comparable ethical standards. As this study utilized the Delphi method for expert consultation and did not involve the collection of any patient data, medical records, or biological samples, the requirement for obtaining informed consent from the expert participants was formally waived by the ethics committee.

### 2.2. Research process

#### 2.2.1. Literature review and current state analysis

The initial phase involved a comprehensive systematic review of the literature to establish the theoretical foundation and identify existing performance indicators used in medical laboratory evaluation. The search strategy employed multiple electronic databases including PubMed, Web of Science, Scopus, and Google Scholar, covering publications from January 2019 to December 2024. Search terms were carefully constructed using Boolean operators and included combinations of “balanced scorecard,” “medical laboratory,” “performance evaluation,” “research management,” “quality indicators,” and related synonyms. The search strategy was designed to capture both theoretical frameworks and empirical applications of performance measurement in healthcare laboratories.^[22,23]^ The literature screening process followed Preferred Reporting Items for Systematic Reviews and Meta-Analyses guidelines, with initial screening based on titles and abstracts, followed by full-text review of potentially relevant articles. Inclusion criteria encompassed: peer-reviewed articles published in English, studies addressing performance evaluation in healthcare or research settings, articles discussing BSC implementation or adaptation, and papers presenting validated indicator systems or frameworks. Exclusion criteria included non-English publications, conference abstracts without full papers, opinion pieces without empirical data, and studies focused exclusively on clinical diagnostics without research components. Two independent reviewers conducted the screening process, with disagreements resolved through discussion and consultation with a third reviewer when necessary. Data extraction focused on identifying performance indicators, measurement methodologies, implementation challenges, and reported outcomes of evaluation systems.

#### 2.2.2. Initial indicator framework development

Based on the systematic literature review findings and analysis of current evaluation practices in medical laboratories, an initial indicator framework was constructed following BSC principles. The development process involved mapping identified indicators to the 4 BSC perspectives while considering the unique characteristics of medical research laboratories. The financial perspective incorporated traditional metrics such as research funding, cost efficiency, and resource utilization, adapted to reflect the nonprofit nature of most medical laboratories. The customer/service perspective was reconceptualized to encompass multiple stakeholder groups including clinicians, patients, research collaborators, and regulatory bodies. The internal process perspective focused on research productivity, quality management, innovation capacity, and operational efficiency. The learning and growth perspective emphasized human capital development, knowledge management, technological advancement, and organizational culture. The initial framework underwent iterative refinement through research team discussions and consultation with laboratory management professionals. Each proposed indicator was evaluated for relevance, measurability, accessibility of data, and strategic importance. The team conducted a gap analysis to identify areas not adequately covered by existing indicators and developed new metrics to address these gaps. Special attention was paid to ensuring balance across the 4 perspectives and incorporating both leading and lagging indicators. The resulting preliminary framework consisted of 4 primary indicators aligned with BSC perspectives, 14 secondary indicators representing key performance domains, and 35 tertiary indicators providing specific measurable metrics.

#### 2.2.3. Expert panel selection and delphi consultation

The Delphi method was employed to achieve expert consensus on the proposed indicator system through structured, iterative consultation rounds.^[24]^ Expert selection followed purposive sampling principles to ensure diverse perspectives and comprehensive domain expertise. Inclusion criteria for expert panel members included: minimum of 10 years of experience in medical laboratory management or research administration, senior professional title (associate professor, senior researcher, or equivalent administrative position), demonstrated expertise in performance evaluation or quality management as evidenced by publications or professional certifications, current or recent involvement in strategic planning or policy development for medical laboratories, and willingness to participate in multiple consultation rounds. The recruitment process initially identified 25 potential experts through professional networks, literature authorship, and institutional recommendations. After screening for eligibility and obtaining informed consent, 17 experts agreed to participate, representing diverse backgrounds including laboratory directors (n = 5), research administrators (n = 4), quality management specialists (n = 3), healthcare policy experts (n = 3), and academic researchers specializing in performance evaluation (n = 2). The geographic distribution included experts from academic medical centers, government research institutes, and independent laboratories across multiple regions, ensuring broad representativeness.

The first round of Delphi consultation was conducted through an electronic questionnaire distributed via a secure online platform in March 2024. The questionnaire presented the initial indicator framework with detailed descriptions of each indicator, measurement methods, and data sources. Experts were asked to rate each indicator on a 5-point Likert scale for importance (1 = not important to 5 = extremely important) and feasibility (1 = not feasible to 5 = highly feasible). Additionally, open-ended questions solicited suggestions for indicator modification, addition, or deletion, as well as comments on the overall framework structure. The response rate for the first round was 100% (17/17), with all questionnaires completed within the designated 3-week period. Following the first round, quantitative responses were analyzed using descriptive statistics to calculate mean scores, standard deviations, and coefficients of variation for each indicator. Indicators with mean importance scores below 3.5 or feasibility scores below 3.0 were flagged for potential removal or substantial modification. Qualitative feedback was analyzed using thematic analysis to identify common concerns, suggestions, and areas of disagreement among experts. Based on this analysis, the research team revised the indicator framework, consolidating redundant indicators, clarifying ambiguous definitions, and incorporating suggested new metrics that received strong support.

The second round of Delphi consultation was conducted in May 2024, presenting the revised framework along with aggregated results from the first round. This feedback included statistical summaries of ratings and anonymized representative comments to facilitate informed reconsideration of positions. Experts were asked to rerate all indicators and provide additional comments on the revisions. The response rate remained at 100% for the second round. Analysis revealed substantial improvement in consensus, with reduced coefficients of variation and increased mean scores for retained indicators. Kendall coefficient of concordance (*W*) was calculated to assess the degree of agreement among experts, yielding *W* = 0.812 (*P* < .001), indicating strong consensus.^[25]^

#### 2.2.4. Indicator screening and optimization

The indicator screening and optimization process involved systematic evaluation of all proposed metrics based on multiple criteria to ensure the final framework’s validity, reliability, and practical utility. Quantitative screening criteria included minimum threshold scores from the Delphi consultation (importance ≥ 4.0, feasibility ≥ 3.5), coefficient of variation < 0.25 indicating acceptable agreement, and correlation analysis to identify redundant indicators with correlation coefficients > 0.8. Qualitative screening involved content analysis of expert comments to identify conceptual overlaps, implementation barriers, and measurement challenges. The optimization process focused on achieving parsimony while maintaining comprehensive coverage of performance domains. Indicators that measured similar constructs were consolidated into composite metrics where appropriate. For instance, multiple publication-related metrics were combined into a weighted publication index that considered both quantity and quality factors. The research team also ensured that each BSC perspective retained sufficient indicators to provide meaningful assessment while avoiding excessive complexity that could hinder implementation. Special attention was paid to maintaining balance between objective quantitative metrics and subjective qualitative assessments, recognizing that both types of information are valuable for comprehensive performance evaluation. Data availability and collection feasibility were critical considerations in the optimization process. Each indicator was evaluated for data source accessibility, collection burden, and potential for automation or integration with existing information systems. Indicators requiring extensive manual data collection or specialized expertise for interpretation were modified or replaced with more practical alternatives where possible. The team also considered temporal aspects, ensuring a mix of indicators that could provide both real-time monitoring capabilities and longer-term strategic insights.

#### 2.2.5. Weight determination using AHP

The AHP was employed to determine the relative weights of indicators within the hierarchical framework, providing a systematic approach to quantifying the relative importance of different performance dimensions.^[26]^ The AHP methodology began with constructing the hierarchical structure comprising the overall goal (comprehensive performance evaluation), primary indicators (BSC perspectives), secondary indicators (performance domains), and tertiary indicators (specific metrics). This hierarchical decomposition facilitated systematic comparison and weight derivation at each level.

Pairwise comparison matrices were developed for each level of the hierarchy, with experts asked to compare the relative importance of indicators using Saaty 9-point scale (1 = equal importance to 9 = extreme importance). To ensure consistency and reduce cognitive burden, comparisons were structured systematically, with experts first comparing primary indicators, then secondary indicators within each primary category, and finally tertiary indicators within each secondary category. A subset of 10 experts from the original Delphi panel who demonstrated strong engagement and expertise participated in the AHP process, conducted through face-to-face workshops supplemented by online consultations for clarification and validation. The judgment matrices were analyzed using eigenvalue methods to derive priority weights, with the principal eigenvector representing the relative weights of compared elements. Consistency ratios (CR) were calculated for each matrix to assess the logical consistency of expert judgments, with CR < 0.10 considered acceptable according to established AHP guidelines. Initial analysis revealed several matrices with CR > 0.10, prompting focused discussions with experts to identify and resolve inconsistencies in their comparisons. Through iterative refinement, all matrices achieved acceptable consistency levels, with final CR values ranging from 0.042 to 0.089. The aggregation of individual expert judgments followed the geometric mean method, recommended for group decision-making in AHP applications. This approach preserves the reciprocal property of comparison matrices and provides a balanced representation of diverse expert opinions. Sensitivity analysis was conducted to assess the robustness of derived weights to variations in expert judgments, with results indicating stable weight distributions across reasonable perturbations. The final weights reflected both the strategic importance of different performance dimensions and practical considerations for implementation in medical laboratory settings.

### 2.3. Data analysis tools and quality assurance

Statistical analyses were performed using SPSS version 26.0 for descriptive statistics, reliability testing, and agreement analysis, while Expert Choice software was utilized for AHP calculations and sensitivity analysis. All quantitative data underwent verification for accuracy and completeness before analysis, with missing data addressed through follow-up with respondents where possible. Qualitative data from open-ended responses were managed using NVivo software to facilitate systematic coding and thematic analysis. Inter-rater reliability for qualitative coding was assessed using Cohen kappa, achieving κ = 0.84, indicating substantial agreement between coders. Quality assurance measures were implemented throughout the research process to ensure methodological rigor and validity of results. These included maintaining detailed documentation of all methodological decisions and modifications, conducting regular team meetings to review progress and resolve challenges, obtaining expert feedback on interim results before proceeding to subsequent phases, and performing member checking by sharing synthesized results with participating experts for validation. The research team also established an audit trail documenting the evolution of the indicator framework from initial conception through final validation, enabling transparency and reproducibility of the research process.

## 3. Results

### 3.1. Initial indicator framework construction

The systematic literature review and analysis yielded a comprehensive initial framework comprising 35 indicators distributed across the 4 BSC perspectives. The financial perspective included 8 indicators focusing on research funding acquisition, resource utilization efficiency, and cost-effectiveness measures. The customer/service perspective encompassed 9 indicators addressing stakeholder satisfaction, research impact on clinical practice, and collaborative partnerships. The internal process perspective contained 10 indicators covering research productivity, quality management systems, and innovation processes. The learning and growth perspective included 8 indicators related to human capital development, knowledge management, and organizational capacity building. This initial framework is presented in Table 1.

**Table 1:** Initial performance evaluation indicator framework for medical laboratory research management.

Primary Indicator	Secondary Indicator	Tertiary Indicator	Initial Mean Score
Financial Perspective	Research Funding	Total research grants received	4.65
		Grant success rate	4.41
		Diversity of funding sources	4.12
	Resource Efficiency	Cost per research output	3.88
		Equipment utilization rate	4.06
	Revenue Generation	Technology transfer income	3.94
		Clinical service revenue	3.76
		Training program revenue	3.53
Customer/Service Perspective	Clinical Impact	Clinical guideline contributions	4.71
		Patient outcome improvements	4.82
	Stakeholder Satisfaction	Clinician satisfaction score	4.35
		Research partner satisfaction	4.29
		Student/trainee satisfaction	4.18
	Collaboration Network	Number of active collaborations	4.47
		International partnership index	4.24
	External Recognition	Awards and honors received	3.88
		Media coverage index	3.41
Internal Process Perspective	Research Productivity	Publication output (weighted)	4.76
		Citation impact factor	4.59
		Patent applications filed	4.06
	Quality Management	Laboratory accreditation status	4.88
		Quality control performance	4.71
		Research protocol compliance	4.65
	Innovation Capacity	New methodology development	4.53
		Research portfolio diversity	4.29
	Operational Efficiency	Project completion rate	4.41
		Time to research translation	4.35
Learning & Growth Perspective	Human Capital	Staff qualification index	4.47
		Training hours per researcher	4.24
		Staff retention rate	4.18
	Knowledge Management	Knowledge sharing activities	4.35
		Internal seminar frequency	3.94
		Database/repository development	4.41
	Technology Infrastructure	IT system capability index	4.29
		Equipment modernization rate	4.53
	Organizational Culture	Innovation climate assessment	4.12

### 3.2. Expert consultation results

The 2 rounds of Delphi consultation achieved high response rates and demonstrated progressive convergence toward consensus. Table 2 summarizes the participation and agreement metrics across both consultation rounds.

**Table 2:** Delphi consultation response rates and coordination coefficients.

Consultation Metrics	Round 1	Round 2
Questionnaires distributed	17	17
Questionnaires returned	17	17
Response rate (%)	100	100
Average completion time (d)	12.3	9.7
Kendall *W* coefficient	0.683	0.812
Chi-square value	394.7	469.5
*P* value	< .001	< .001
Number of indicators modified	-	12
Number of indicators added	-	3
Number of indicators removed	-	7
Final consensus achievement (%)	76.5	94.1

**Figure 1.**
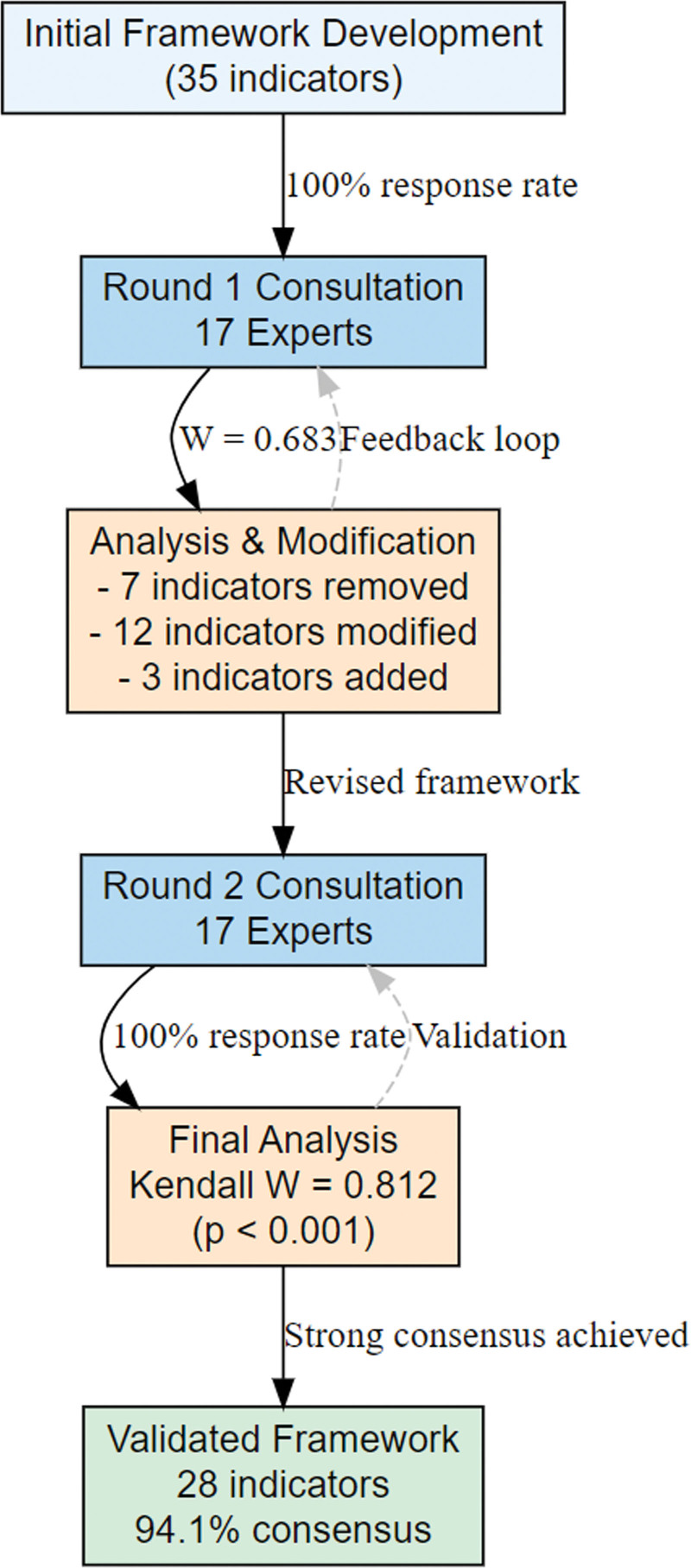
Delphi consultation process flow diagram.

The expert consultation process is illustrated in Figure 1, whichdemonstrates the iterative refinement approach and decision points throughout the Delphi rounds.

### 3.3. Indicator screening and modification

The screening process resulted in significant refinement of the indicator framework based on expert feedback and statistical analysis. Table 3 presents the screening results and retention rates for indicators across different categories.

**Table 3:** Indicator screening process and retention rates.

Indicator Category	Initial Number	After Round 1	After Round 2	Final Number	Retention Rate (%)
Financial Perspective	8	7	7	7	87.5
Customer/Service	9	8	7	7	77.8
Internal Process	10	9	8	8	80.0
Learning & Growth	8	7	6	6	75.0
Total Indicators	35	31	28	28	80.0
Primary Indicators	4	4	4	4	100.0
Secondary Indicators	14	13	12	12	85.7
Tertiary Indicators	35	31	28	28	80.0

The modification process involved consolidating redundant indicators, clarifying measurement definitions, and incorporating new metrics suggested by experts. Notable changes included combining multiple publication metrics into a comprehensive research output index, adding indicators for translational research impact, and removing indicators with low feasibility scores such as detailed cost accounting metrics that many laboratories lack systems to track accurately.

### 3.4. Final performance evaluation indicator system

The validated performance evaluation framework represents a comprehensive yet practical system for assessing medical laboratory research management performance. Table 4 presents the final indicator system with calculated weights from the AHP analysis.

**Table 4:** Final BSC-based performance evaluation indicator system with weights.

Primary Indicator (Weight)	Secondary Indicator (Local Weight)	Tertiary Indicator	Global Weight
Financial Perspective (0.278)	Research Funding (0.425)	Annual research grant total	0.0329
		Grant application success rate	0.0281
		Funding source diversity index	0.0247
		Research funding growth rate	0.0318
	Resource Utilization (0.337)	Cost-effectiveness ratio	0.0312
		Core facility utilization rate	0.0287
		Resource sharing efficiency	0.0268
	Financial Sustainability (0.238)	Revenue diversification index	0.0221
		Budget variance rate	0.0198
		Return on research investment	0.0243
Customer/Service Perspective (0.245)	Clinical Translation (0.382)	Clinical practice guidelines influenced	0.0287
		Patient care improvements documented	0.0312
		Diagnostic/therapeutic innovations adopted	0.0268
	Stakeholder Engagement (0.346)	Healthcare provider satisfaction index	0.0254
		Research collaboration satisfaction	0.0238
		Patient/public engagement score	0.0221
	Academic Impact (0.272)	Educational program quality rating	0.0198
		Graduate student outcomes	0.0187
		Continuing education participation	0.0165
Internal Process Perspective (0.245)	Research Excellence (0.418)	Weighted publication index	0.0321
		Citation impact normalized score	0.0298
		Research innovation index	0.0265
	Quality Systems (0.341)	Accreditation compliance rate	0.0278
		Quality incident rate (inverse)	0.0234
		Process standardization index	0.0223
	Operational Excellence (0.241)	Project milestone achievement rate	0.0198
		Research cycle time efficiency	0.0176
		Data management maturity score	0.0165
Learning & Growth Perspective (0.232)	Human Capital Development (0.397)	Professional development index	0.0276
		Research competency assessment score	0.0254
		Talent retention rate	0.0234
	Knowledge Assets (0.352)	Knowledge transfer activity index	0.0232
		Intellectual property portfolio value	0.0221
		Research database utility score	0.0198
	Innovation Culture (0.251)	Innovation climate survey score	0.0176
		Cross-functional collaboration index	0.0154
		Continuous improvement participation	0.0143

BSC = balanced scorecard.

**Figure 2.**
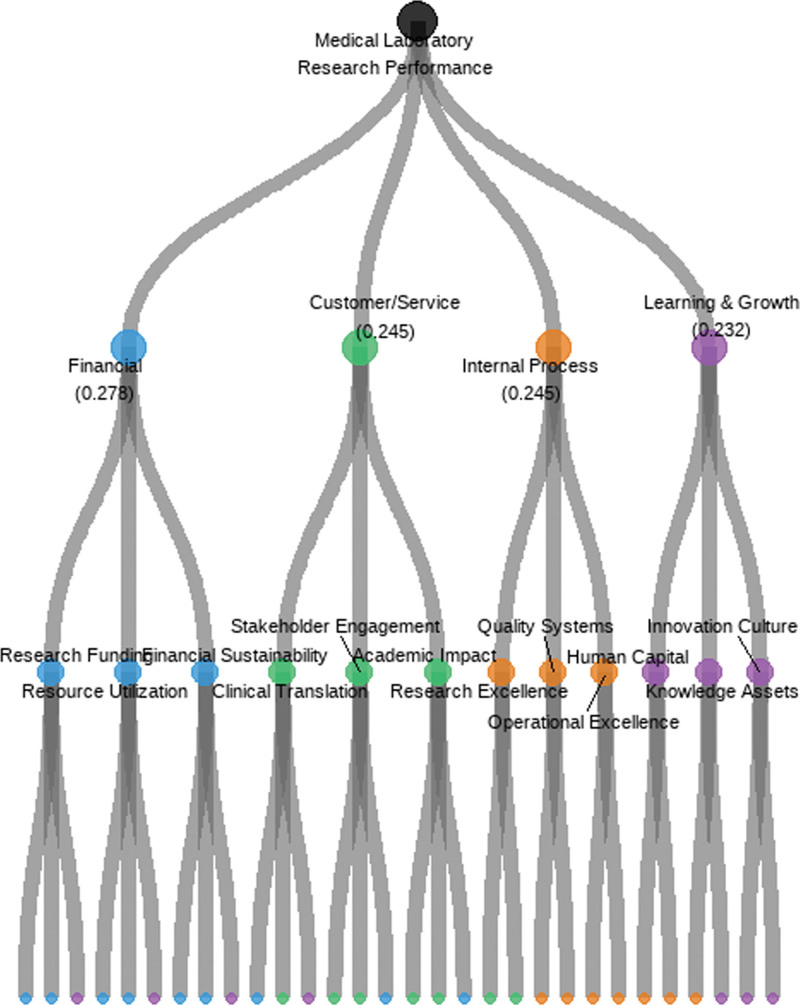
Hierarchical structure of BSC-based performance evaluation framework. A hierarchical diagram showing 4 main branches representing the BSC perspectives (financial, customer/service, internal process, learning & growth) emanating from a central node labeled “Medical Laboratory Research Performance.” Each branch further divides into secondary indicators (3–4 per perspective) and tertiary indicators (2–3 per secondary indicator). The diagram uses varying line weights to represent the relative importance of connections, with thicker lines indicating higher weights. Color coding differentiates the 4 perspectives: financial (blue), customer/service (green), internal process (orange), and learning & growth (purple). BSC = balance scorecard.

The hierarchical structure of the final indicator system is visualized in Figure 2, illustrating the relationships between different levels of indicators and their relative contributions to overall performance assessment.

### 3.5. Weight distribution analysis

The AHP analysis revealed differentiated importance across the 4 BSC perspectives and their constituent indicators. Table 5 provides a detailed breakdown of weight distributions at each hierarchical level.

**Table 5:** Weight distribution across BSC dimensions and indicator levels.

Analysis Level	Indicator Category	Weight Range	Mean Weight	SD	CR
Primary (Level 1)	BSC Perspectives	0.232–0.278	0.250	0.019	0.089
	Financial	0.278	-	-	-
	Customer/Service	0.245	-	-	-
	Internal Process	0.245	-	-	-
	Learning & Growth	0.232	-	-	-
Secondary (Level 2)	Within Financial	0.238–0.425	0.333	0.076	0.074
	Within Customer	0.272–0.382	0.333	0.046	0.068
	Within Internal	0.241–0.418	0.333	0.074	0.071
	Within Learning	0.251–0.397	0.333	0.061	0.065
Tertiary (Level 3)	All Indicators	0.0143–0.0329	0.0238	0.0056	0.042–0.082
	Top 5 Weighted	0.0312–0.0329	0.0320	0.0007	-
	Bottom 5 Weighted	0.0143–0.0165	0.0155	0.0009	-

BSC = balanced scorecard, CR = consistency ratio, SD = standard deviation.

The weight distribution analysis demonstrates relatively balanced importance across BSC perspectives, with slight emphasis on financial sustainability and internal processes, reflecting the dual pressures of resource constraints and quality requirements in medical laboratory research management.

**Figure 3.**
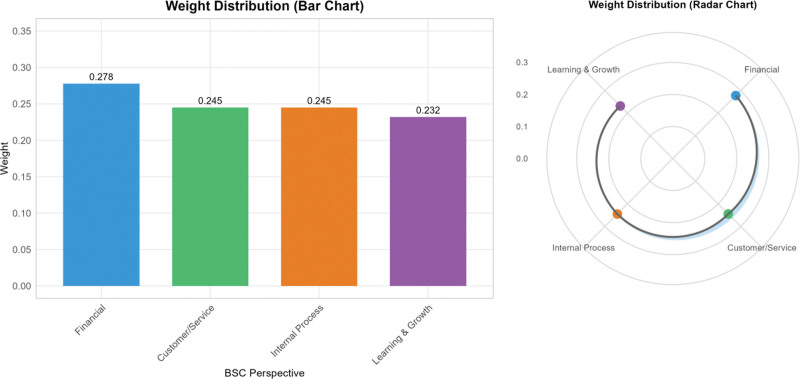
Comparative weight distribution across BSC dimensions. BSC = balance scorecard.

Figure 3 presents a visual comparison of dimension weights using both bar chart and radar chart representations to illustrate the balanced yet differentiated emphasis across performance dimensions.

## 4. Discussion

The successful development and validation of a BSC-based performance evaluation framework for biomedical research laboratory management represents a significant advancement in healthcare performance measurement. The framework’s balanced distribution of weights across the 4 BSC perspectives, with financial (0.278), customer/service (0.245), internal process (0.245), and learning and growth (0.232) dimensions receiving relatively equal emphasis, demonstrates the successful adaptation of BSC principles to the unique context of medical research laboratories. This finding aligns with recent research emphasizing the importance of multidimensional performance assessment in healthcare organizations, while extending previous work by specifically addressing the research management domain.^[27,28]^

The high level of expert consensus achieved (*W* = 0.812, *P* < .001) validates the framework’s content validity and practical relevance. This consensus level exceeds the threshold of 0.7 generally considered indicative of strong agreement in Delphi studies, suggesting that the proposed indicators resonate with the diverse perspectives of laboratory managers, researchers, and policy experts.^[29]^ The iterative refinement process, which resulted in a 20% reduction in the number of indicators from the initial framework, demonstrates the value of expert consultation in achieving parsimony without sacrificing comprehensiveness. This finding contributes to the methodological literature on indicator development by demonstrating the effectiveness of combining systematic review, Delphi consultation, and AHP in creating robust evaluation frameworks.

The framework’s emphasis on research translation and stakeholder engagement, as evidenced by the high weights assigned to clinical translation indicators (0.0287–0.0312) and stakeholder satisfaction metrics (0.0221–0.0254), reflects the evolving mission of medical laboratories from purely research-focused entities to integrated components of the healthcare innovation ecosystem. This orientation toward translational impact distinguishes our framework from traditional academic performance metrics that primarily emphasize publication outputs and grant funding. The inclusion of specific indicators for patient care improvements and clinical guideline contributions addresses the growing demand for demonstrable healthcare value from research investments, consistent with the value-based healthcare paradigm increasingly adopted worldwide.^[30]^

When compared to existing performance evaluation systems for medical laboratories, our BSC-based framework offers several distinctive advantages while addressing identified limitations in current approaches. Traditional laboratory evaluation systems have predominantly focused on operational efficiency and quality control metrics, as exemplified by ISO 15189 accreditation standards and clinical laboratory improvement amendments requirements. While these frameworks provide essential quality assurance, they offer limited guidance for research performance assessment and strategic management. Our framework complements these operational standards by incorporating research-specific metrics while maintaining alignment with quality management principles through the internal process perspective.

Recent studies have proposed various performance indicator systems for healthcare laboratories, though most focus primarily on clinical service delivery rather than research management. The study by Alvarez et al (2019) developed a BSC for clinical laboratories that emphasized diagnostic turnaround times, test accuracy, and cost per test, with limited attention to research activities.^[12]^ In contrast, our framework explicitly balances clinical service metrics with research productivity indicators, recognizing the dual mission of key medical laboratories. The inclusion of innovation capacity measures and knowledge management indicators addresses gaps identified in previous frameworks that failed to capture the dynamic, knowledge-intensive nature of medical research.

The weight distribution in our framework differs notably from business-oriented BSC applications, which typically assign higher weights to financial perspectives. Healthcare-specific BSC implementations have shown varied weight distributions, with some emphasizing customer satisfaction and others prioritizing internal processes. Our relatively balanced weighting scheme (ranging from 0.232–0.278 across perspectives) reflects the unique position of medical laboratories as entities that must balance financial sustainability with public service obligations, scientific excellence with clinical relevance, and current performance with future capability development. This balanced approach aligns with recommendations from recent systematic reviews suggesting that healthcare BSC implementations should avoid overemphasis on any single perspective.^[31]^

International comparisons reveal variations in evaluation priorities across healthcare systems, with European frameworks emphasizing quality and safety, North American systems prioritizing efficiency and financial performance, and Asian systems focusing on innovation metrics. Our framework synthesizes these diverse emphases by incorporating indicators addressing quality, efficiency, innovation, and stakeholder value, enabling cross-context applicability with local adaptation through weight adjustments. Practical implementation requires consideration of organizational readiness, data infrastructure, and change management. Biomedical research laboratories vary significantly in measurement capabilities, from sophisticated business intelligence systems to manual data collection. Some indicators, such as stakeholder satisfaction and innovation climate, may require new data collection mechanisms that could be challenging in resource-limited settings. The framework’s hierarchical structure enables phased implementation, starting with primary and secondary indicators before expanding to tertiary metrics as capabilities mature. For smaller or less-resourced laboratories, a simplified version focusing on core indicators could be developed in future adaptations. Data availability and quality present critical challenges. While publication counts and grant totals are readily accessible, stakeholder satisfaction and innovation climate assessments require new collection mechanisms. Laboratories need integrated information systems capturing performance data from research management, financial, HR, and quality platforms. Standardized data definitions and protocols ensure consistency across time periods and units. Beyond measurement, the framework supports strategic planning, resource allocation, and continuous improvement. Laboratory directors can use the BSC to communicate priorities and align organizational efforts, with explicit performance dimension linkages helping staff understand their contributions’ impact. Regular metric review in management meetings ensures performance data inform decision-making rather than merely serving compliance requirements.

This study has several limitations that warrant acknowledgments. The expert panel of 17 participants, while professionally diverse, was geographically concentrated in developed healthcare systems and may not fully represent global biomedical research laboratory contexts, particularly resource-constrained settings with different performance priorities. Future studies should validate and adapt this framework in low-resource settings to enhance its global applicability. The cross-sectional development approach may not adequately capture evolving performance evaluation needs, especially given recent lessons from the COVID-19 pandemic regarding surge capacity, rapid diagnostic development, and crisis communication: areas not fully addressed in the current framework. Additionally, the institutional-level focus may obscure individual or team-level performance variations within laboratories, suggesting the need for multi-level evaluation approaches. Finally, while expert consensus provides face and content validity, future longitudinal studies are warranted to establish predictive validity and demonstrate that laboratories with higher BSC scores achieve better long-term outcomes, while also identifying potential unintended consequences such as metric fixation or neglect of unmeasured dimensions.

## 5. Conclusion

This study successfully developed a BSC-based performance evaluation framework for medical laboratory research management, comprising 4 primary, 12 secondary, and 28 tertiary indicators that balance financial and nonfinancial dimensions while incorporating research translation and stakeholder engagement metrics. The framework achieved high expert consensus through Delphi consultation and AHP analysis, validating its content validity and practical applicability. By adapting BSC principles to medical laboratory contexts, it bridges management theory and healthcare research practice, providing administrators with a comprehensive assessment tool. As laboratories face increasing pressure to demonstrate value, this framework offers a structured approach to performance evaluation while maintaining focus on scientific advancement.

## Acknowledgments

The authors would like to thank all the experts who participated in the Delphi consultation and AHP process for their valuable time and insights. We confirm that all individuals named in this acknowledgments section have provided consent to be mentioned.

## Author contributions

**Conceptualization:** Qi Wang.

**Formal analysis:** Bing Zhou.

**Project administration:** Jia Niu.

**Resources:** Bing Zhou.

**Software:** Jia Niu.

**Visualization:** Qi Wang.

**Writing – original draft:** Bing Zhou, Jia Niu.

**Writing – review & editing:** Bing Zhou, Qi Wang.
